# Platelet Microparticles and miRNA Transfer in Cancer Progression: Many Targets, Modes of Action, and Effects Across Cancer Stages

**DOI:** 10.3389/fcvm.2018.00013

**Published:** 2018-02-28

**Authors:** Sophia Lazar, Lawrence E. Goldfinger

**Affiliations:** ^1^The Sol Sherry Thrombosis Research Center, Department of Anatomy and Cell Biology, Lewis Katz School of Medicine, Temple University, Philadelphia, PA, United States; ^2^Cancer Biology Program, Fox Chase Cancer Center, Philadelphia, PA, United States

**Keywords:** platelets, miRNAs, cancer, microparticles, tumor growth

## Abstract

Platelet-derived microparticles (PMPs) have long been known to increase in circulation in the presence of cancer, and have been considered to be cancer promoting by multiple mechanisms including shrouding of circulating tumor cells allowing immune evasion, inducing a procoagulant state associated with increased risk for venous thromboembolic events in cancer patients, and supporting metastatic dissemination by establishment of niches for anchorage of circulating tumor cells. These modes of PMP-enhanced progression of late stage cancer are generally based on the adhesive and procoagulant surfaces of PMPs. However, it is now clear that PMPs can also act as intercellular signaling vesicles, by fusion with target cells and transfer of a broad array of platelet-derived molecular contents including growth factors, angiogenic modulators, second messengers, lipids, and nucleic acids. It is also now well established that PMPs are major repositories of microRNAs (miRNAs). In recent years, new roles of PMPs in cancer have begun emerging, primarily reflecting their ability to transfer miRNA contents and modulate gene expression in target cells, allowing PMPs to affect cancer development at many stages. PMPs have been shown to interact with and transfer miRNAs to various blood vascular cells including endothelium, macrophages and neutrophils. As each of these contributes to cancer progression, PMP-mediated miRNA transfer can affect immune response, NETosis, tumor angiogenesis, and likely other cancer-associated processes. Recently, PMP miRNA transfer was found to suppress primary tumor growth, via PMP infiltration in solid tumors, anchorage to tumor cells and direct miRNA transfer, resulting in tumor cell gene suppression and inhibition of tumor growth. This mini-review will summarize current knowledge of PMP-miRNA interactions with cancer-associated cells and effects in cancer progression, and will indicate new research directions for understanding platelet-cancer interactions.

## Introduction

Platelets, which are derived from megakaryocytes, are blood-borne cell fragments that are the primary cellular agents in hemostasis and thrombosis. Vascular injury or thrombogenic stimuli activate platelet receptors, promoting platelet granule secretion, coagulation, and platelet crosslinking of fibrin leading to thrombus formation (1). Activated platelets also release platelet-derived microparticles (PMPs): extracellular vesicles formed by outward blebbing of the platelet plasma membrane followed by scission, trapping platelet-derived cytosolic material, which account for up to 90% of circulating microparticles (MPs) (2). The adhesive and pro-coagulant nature of PMPs, due to their surface expression of phosphatidylserine (PS) and other platelet antigens, contributes to the prothrombotic state often associated with pathologies which display increased levels of circulating PMPs, including cancer (3). PMPs also play important roles in long-range cell-to-cell communication as they contain many of the same proteins, lipids, and oligonucleotides as their platelet progenitors. In particular, PMPs are enriched in microRNAs (miRNAs) derived from circulating platelets (4). MiRNAs are small non-coding RNAs that suppress mRNA translation through multiple mechanisms. Within the nucleus of megakaryocytes, long primary RNAs harboring tandem miRNAs transcribed by RNA polymerase II are processed by Drosha and DGCR8 into short hairpin pre-miRNAs, which are subsequently exported to the cytoplasm (5). These cytoplasmic pre-miRNAs are inherited by maturing platelets along with Dicer1, the endonuclease responsible for generation hairpin cleavage to generate mature miRNA, consisting of an approximately 22 nt long RNA duplex; thus, platelets are enriched in pre-miRNAs and mature miRNAs (6–8). These platelet-derived miRNAs are packaged into PMPs and constitute a major fraction of the platelet content released in PMPS (9). PMPs have long been known to increase in circulation in cancer patients (10), which has been associated with poor cancer prognosis (11). However, our understanding of the PMP-cancer axis is still evolving; it is now clear that PMPs and their entrapped miRNAs also have tumor suppressive roles, as we have recently found with respect to growth of primary solid tumors (12). Thus, the roles of PMPs and platelet miRNAs in cancer progression are multi-faceted and not yet completely understood.

### Cancer-Promoting Roles of PMPs

Activated platelets, as well as PMPs, are known to play important roles in cancer progression. The presence of cancer also promotes platelet activation, creating the so-called platelet-cancer loop. Indeed, cancer patients are at increased risk for platelet-driven venous thromboembolism, which is a significant contributor to morbidity and mortality in cancer patients, depending on tissue type; pancreatic and lung cancer patients are at especially high risk (13). This prothrombotic state in cancer is due not only to increased platelet activation, but also to the concomitant increase in PS-positive PMPs which further enhance coagulation (11, 14). Increases in circulating PMPs vary by cancer type but generally appear to increase with advanced cancer stage. For example, PMP levels in patients with myeloproliferative neoplasms were roughly doubled compared to healthy subjects, up to four times in colorectal and oral cancer patients, as high as 10 times higher in breast cancer, and up to 35 times higher in stage IV gastic cancer; in each cohort the highest levels were found in advanced stage cancer and in most cases were associated with the presence of distal metastases (15–19). Platelets can shroud blood-borne tumor cells, protecting them from immune detection (20), suggesting that PMPs may play a similar role. This tumor-shrouding property of platelets may also be supported by PMPs, as PMP membranes contain the same proteins as platelet surfaces. However, further studies are needed to determine if PMPs truly act in this fashion to promote cancer development.

Though platelets have long been known to play a key role in metastatic dissemination, involvement of PMPs, through multiple mechanisms, has only recently been shown. Matrix metalloproteinases (MMPs) are the principal enzymes involved in degradation of the extracellular matrix (ECM), preceding both intravasation and angiogenesis (21). PMPs have been shown to promote secretion of MMP-2, specifically in prostate cancer cells, thereby promoting vessel invasion (22). A similar role for PMPs has been demonstrated with lung cancer cells *in vitro*, where PMPs were shown to transfer integrin CD41 to cancer cells as well as to promote surface expression of membrane type 1-MMPs (23). Interestingly, the same study also reported that PMP-associated Lewis Lung Carcinoma cells produced tumor foci with increased metastatic potential, indicating the pro-metastatic nature of these molecules (23). In addition, the pro-coagulant surface of PMPs facilitates docking of metastatic tumor cells to distal sites, thereby facilitating establishment of new nodes (24, 25); this may go a long way to explain the correlation between increased PMPs and poor prognosis in cancer.

Both platelets and PMPs have also been shown to play roles in neoangiogenesis, a process required for both growth of primary solid tumors and metastasis. PMPs promote endothelial proliferation *in vitro*, due in part to their lipid components and surface receptors (26). The ability of PMPs to prompt an angiogenic response both *in vitro* and *in vivo* has been attributed to their harboring cytokines such as vascular endothelial growth factor (VEGF), basic fibroblast growth factor, and platelet-derived growth factor (27). Alternatively, PMPs may exert their pro-angiogenic effects by binding a variety of signaling molecules including tissue factor (TF), platelet-activating factor, and VEGF (28). While is it now clear that PMPs are intimately tied to both metastatic dissemination of tumor cells and angiogenesis, tumor angiogenesis has not been thoroughly explored in this context; moreover, these effects have been primarily attributed to the surface properties of PMPs, and thus putative roles of their cargo have yet to be elucidated.

### PMPs as Intercellular Signaling Vesicles

PMPs encapsulate growth factors, angiogenic modulators, and nucleic acids derived from platelets (29). Importantly, PMPs also contain platelet-derived bioactive molecules, such as sphingosine 1-phosphate (S1P) and arachidonic acid (AA) (30). Due to their small size, ability to travel long distances through the blood stream, and capacity to fuse with or be internalized by target cells, PMPs have been proposed to play important physiological roles as intercellular signaling vesicles (30). Specific effects of molecular transfer via PMPs may be dependent on target cell type; for example, PMP exposure can variably suppress mitotic and migration signaling genes in endothelial cells, promote adhesion and proliferation of some normal and transformed blood cells such as increasing monocyte-endothelial interaction, or induce chemotactic motility of monocytes (9, 29, 31, 32).

Circulating PMPs are enriched in miRNAs (33), which are conserved and potent regulators of gene expression. Importantly, miRNA content of PMPs appears to constitute subsets of miRNA cohorts in platelets, indicating that miRNAs are actively selected and packaged into PMPs for secretion rather than stochastically incorporated (34). Analysis of patient samples has shown that circulating PMP miRNA content is altered during various pathologies, implying they may function as potential biomarkers for disease as well as platelet activation (34, 35). Purified PMPs can transfer their miRNAs to recipient cells, with particular physiological effects tied to transport of specific miRNAs to distinct cell types. A summary of known PMP-encapsulated miRNAs transferred to different cell types involved in cancer and established or putative effects is listed in Table 1. For example, PMPs released following thrombin activation of platelets are enriched in miR-223. Internalization of PMPs by human umbilical vein endothelial cells (HUVEC), and subsequent transfer of Argonaute 2 (Ago2) · miR-223 complexes results in downregulation of gene expression for targets of miR-223 within the recipient endothelial cells, which in some cases may lead to endothelial apoptosis (9, 36). In contrast, PMP-mediated transfer of the same miRNA to lung cancer cells increases cancer invasiveness by suppression of EPB41L3, a known tumor suppressor (37). Whether these distinctions reflect biased selection of gene targets of particular miRNAs is not clear; next generation sequencing for RNA expression and expanded mapping of miRNA targets is needed to elucidate the full scope of platelet miRNA effects.

**Table 1:** PMP-encapsulated miRNAs and associated roles in cancer

**miRNA**	**Role**	**Cell Type**	**Reference**
miR-223	Suppression of FBXW7 and EFNA1, possibly resulting in apoptosis	HUVEC (endothelial)	(9, 36)
miR-223	Suppression of EPB41L3, leading to increased invasiveness	Lung cancer cells	(37)
miR-126-3p	Increased phagocytic phenotype	Primary human macrophages	(38)
miR-183	Suppress natural killer cell activation, possibly via silencing of DAP12	Natural killer cells	(39, 40)
miR-24	Mitochondrial dysfunction and tumor cell apoptosis, leading to suppression of tumor growth	Lung and colon cancer cells	(12)

miRNAs transferred from PMPs to cancer-associated cells are shown, with target cell type and cellular effects.

### Interactions of PMPs with Blood Vascular Cells and Potential Roles in Cancer

PMPs have been shown to interact with many types of blood vascular cells, including circulating tumor cells, leukocytes, and endothelium. Each of these interactions, coupled with PMP-mediated transfer of miRNAs, may affect functions of the target cells during cancer progression. For example, the effects of transfer of miR-223 to endothelium (9) on tumor angiogenesis has not been investigated. Moreover, other miRNAs are known to be transferred from PMPs to endothelial cells; these have yet to be fully characterized, their mRNA targets have not been identified, and functional outcomes with respect to tumor angiogenesis are still unknown.

PMPs themselves have been shown to be pro-inflammatory, triggering cytokine responses via interleukin-1 signaling. This signaling response appears to be specific to PMPs released from platelets activated via the glycoprotein VI (GPVI) collagen receptor complex, suggesting that PMPs produced following activation of GPVI – typically as a result of vascular damage - may contain a subset of miRNAs specialized to induce such a response (41). However, it is likely that other platelet agonists, and platelet hyperactivation generally, contributes to a pro-inflammatory state in cancer; whether this effect aids in immune anti-tumor function remains to be determined. Additionally, PMPs have been shown to interact with monocytes and modify both macrophage and dendritic cell differentiation, implying that PMPs are able to actively modulate the immune response and regulate inflammation (42). However, the exact mechanisms of these interactions are still unknown.

PMPs released by apoptotic platelets, differing to some degree from those deriving from activated platelets, have been shown to promote monocyte differentiation to M2 macrophages, monocyte-derived phagocytic white cells often found at areas of immune activation (43). More recently it has been shown that primary human macrophages are able to internalize PMPs, a process that delivers functional miR-126-3p, which is then able to alter gene expression and lead to a more phagocytic phenotype (38). It will be very interesting to observe how PMP-miRNA stimulation of this phagocytic phenotype relates to the presence, activity, and tumor growth effects of tumor-associated macrophages. Similarly, PMPs can interact with and suppress function of natural killer (NK) cells – innate immune lymphocytes that attack transformed cells – via suppression of NK activation adapter DAP12, through mechanisms that may include transfer of platelet-derived miR-183 (39). This effect is particularly intriguing as miR-183-mediated knockdown of DAP12 suppresses cytolytic function in tumor-associated NK cells (40); it is tempting to speculate that PMPs may act as major catalysts in this process.

PMPs have the capacity to interact with neutrophils, phagocytic granular immune cells, and PMPS mediate interactions between neutrophils with other leukocytes (44), in part due to PMP surface expression of platelet receptors. Glycoprotein Ib (GPIb) receptor, present on the surface of PMPs, interacting with Mac-1 ligands on leukocytes, supports PMP-neutrophil interaction and neutrophil activation (45). PMPs are also able to transfer glycoprotein IIb/IIIa (GPIIb/IIIa) receptors to neutrophils both following isolation and in whole blood (46). It has been suggested that the transferred GPIIb/IIIA receptors are able to participate in NFkB activation of human neutrophils (46). Internalization of PMPs by neutrophils has been shown to be highly regulated, and to be stimulated during autoimmune inflammatory arthritis due to the presence of 12-lipoxygenase and phospholipase A2-IIA (47, 48). Though activated platelets have been shown to stimulate type I interferon production, NETosis by neutrophils, and activation of dendritic cells and T and B lymphocytes (49), it is not yet know if these effects are mediated via PMP release. Neutrophils are intimately involved in cancer progression, via the above effects but also due to their ability to infiltrate tumors and potentially exert anti-tumor effects. However, tumor-associated neutrophils are, like platelets and PMPs, associated with poor cancer prognosis (50). As with PMP-miRNA-monocyte/macrophage interactions, effects of PMP-miRNAs on neutrophil roles in cancer are only just beginning to be explored.

### Cancer-Suppressive Roles of PMP-miRNAs

While PMPs have historically been viewed as cancer promoting, their ability to transfer miRNA and downregulate gene expression in various cell types suggests that PMPs themselves could have tumor-suppressive roles. Our group recently found that PMPs selectively infiltrate solid tumors, potentially as a result of the enhanced permeation and retention, and PMPs transfer platelet miRNAs to the tumor cells, with tumor suppressive effects *in vitro* and *in vivo* across multiple tumor types (12). PMP-mediated miRNA transfer was associated with tumor cell apoptosis, with platelet-derived miR-24 identified as a key miRNA. RNA targets of PMP-transferred miR-24 include mitochondrial *mt-Nd2*, and miRNA transfer resulted in mitochondrial dysfunction and suppression of tumor growth in a miR-24-dependent manner in lung and colon cancer cells *in vitro* and *in vivo* (12). This mechanism of PMP-miRNA-mediated gene regulation and tumor growth suppression requires further exploration. The mechanisms of PMP tumor infiltration, anchorage to tumor cells, and miRNA transfer, as well as the cohort of transferred miRNAs and global effects on tumor cell gene expression, remain to be fully investigated. Nonetheless, it now appears that platelets themselves may be principally cancer-promoting, while tumor-infiltrating PMPs act as a counterbalance, suppressing cancer development of, at least, primary solid tumors (51).

## Conclusions

The interplay between PMPs, platelet miRNAs and cancer progression is elaborate and multifaceted. On the one hand, many of the platelet-like qualities of PMPs, such as their shared expression of surface antigens, promote cancer progression. Platelets themselves have been shown to promote cancer through a variety of mechanisms, yet similar actions of PMPs are still unfolding. Questions regarding PMP involvement in circulating tumor cell immune evasion and metastatic dissemination remain unanswered. In parallel, PMPs were recently shown to mediate horizontal RNA transfer, adding yet another layer of complexity. As PMPs are able to directly inhibit tumor growth, it is possible that platelets and PMPs compensate for one another in regards to their immediate impact on tumor cells themselves, with platelets stimulating and PMPs inhibiting cancer progression. Conversely, transfer of platelet miRNAs via PMPs to other cell types, such as immune cells, appears to indirectly promote cancer. Available data suggest dual-phase effects, wherein PMPs have antineoplastic roles in primary tumor growth through miRNA transfer, but PMPs support cancer progression at later stages through mostly miRNA-independent mechanisms. Much further research is needed to fully elucidate the ways in which platelets and PMPs both interact with tumor cells and modulate cancer progression.

**Figure 1:**
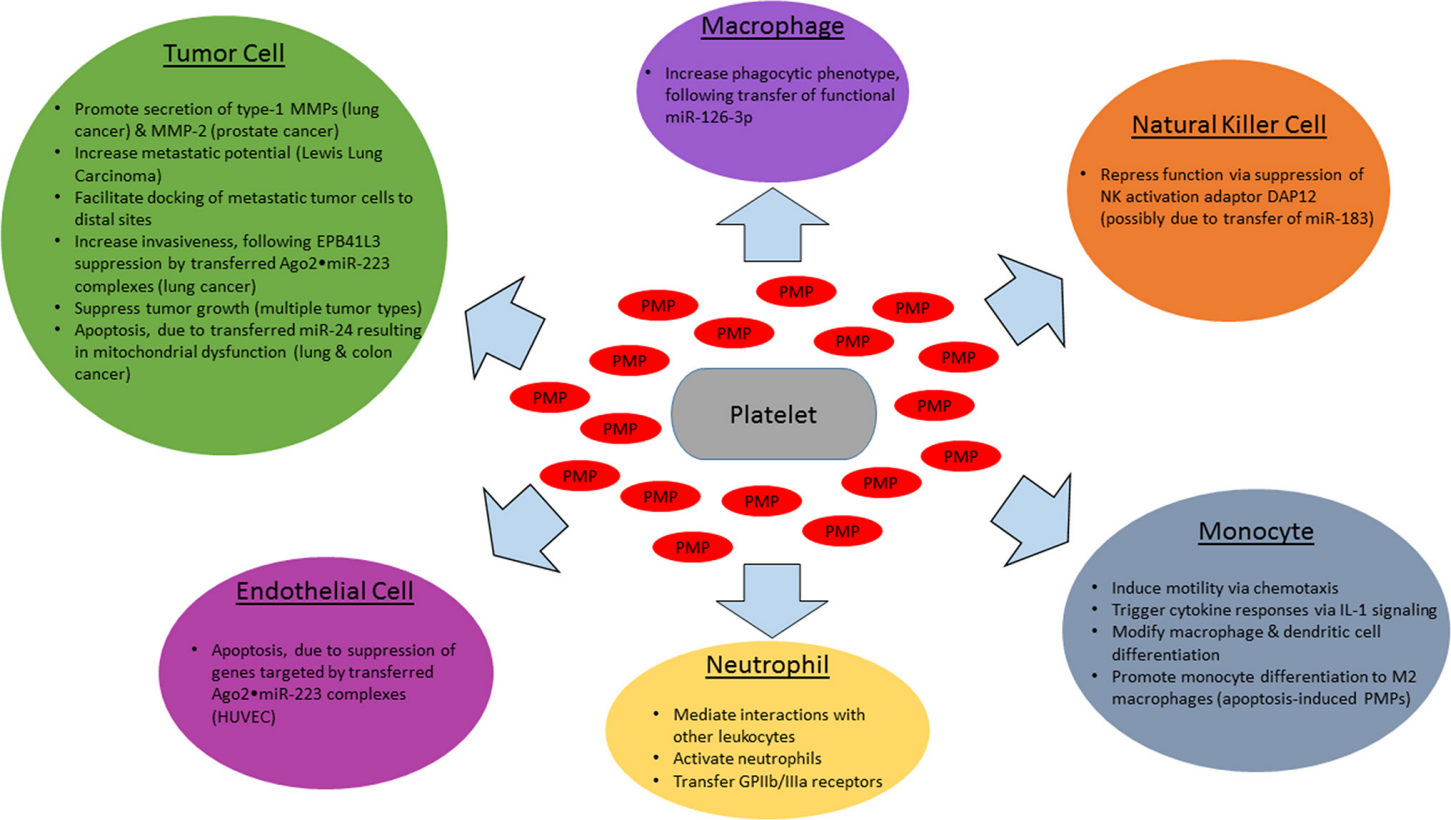
Effects of PMPs and encapsulated miRNAs on cancer-related cell types. Activated platelets release PMPs which interact with various cell types involved in cancer progression, with target cell type-specific effects in cancer. Shown above are the impact of PMP-encapsulated miRNAs (red) on tumor cells (green), endothelial cells (magenta), monocytes (cyan), tissue macrophages (purple), natural killer cells (orange), and neutrophils (yellow).

As it is now clear that PMPs are able to function as signaling vesicles, relaying regulatory miRNAs to a diverse assortment of cell types, a great deal remains to be determined with respect to effects on cancer progression (Figure 1). Specific miRNAs and their mRNA targets have yet to be identified for a majority of tumor cell types, and phenotypic outcomes of mRNA silencing may differ by tumor type. The narrative is even further complicated by PMP-mediated miRNA transfer to vascular cells. Overall, circulating PMPs and the regulatory platelet-derived miRNAs found within them play important roles across many stages of cancer. Elucidating these mechanisms will be of great interest and will reveal potential hot spots for cancer therapeutic targeting.

## Author Contributions

SL and LG wrote and edited the manuscript.

## Conflict of Interest Statement

The authors declare that the research was conducted in the absence of any commercial or financial relationships that could be construed as a potential conflict of interest.
